# Survival and growth patterns of white spruce (*Picea glauca* [Moench] Voss) rangewide provenances and their implications for climate change adaptation

**DOI:** 10.1002/ece3.1100

**Published:** 2014-05-13

**Authors:** Pengxin Lu, William H Parker, Marilyn Cherry, Steve Colombo, William C Parker, Rongzhou Man, Ngaire Roubal

**Affiliations:** 1 Ontario Forest Research Institute, Ontario Ministry of Natural Resources1235 Queen Street East, Sault Ste. Marie, ON, P6A 2E5, Canada; 2 Faculty of Forestry & Forest Environment, Lakehead UniversityThunder Bay, ON, P7B 5E1, Canada; 3 College of Forestry, Oregon State UniversityCorvallis, Oregon, 97331-5704

**Keywords:** Assisted migration, genetic conservation, genetic gain, geographic genetic variation, local adaptation, universal response function

## Abstract

Intraspecific assisted migration (ISAM) through seed transfer during artificial forest regeneration has been suggested as an adaptation strategy to enhance forest resilience and productivity under future climate. In this study, we assessed the risks and benefits of ISAM in white spruce based on long-term and multilocation, rangewide provenance test data. Our results indicate that the adaptive capacity and growth potential of white spruce varied considerably among 245 range-wide provenances sampled across North America; however, the results revealed that local populations could be outperformed by nonlocal ones. Provenances originating from south-central Ontario and southwestern Québec, Canada, close to the southern edge of the species' natural distribution, demonstrated superior growth in more northerly environments compared with local populations and performed much better than populations from western Canada and Alaska, United States. During the 19–28 years between planting and measurement, the southern provenances have not been more susceptible to freezing damage compared with local populations, indicating they have the potential to be used now for the reforestation of more northerly planting sites; based on changing temperature, these seed sources potentially could maintain or increase white spruce productivity at or above historical levels at northern sites. A universal response function (URF), which uses climatic variables to predict provenance performance across field trials, indicated a relatively weak relationship between provenance performance and the climate at provenance origin. Consequently, the URF from this study did not provide information useful to ISAM. The ecological and economic importance of conserving white spruce genetic resources in south-central Ontario and southwestern Québec for use in ISAM is discussed.

## Introduction

Climate change is projected to alter the growing conditions of forest trees considerably (Rahmstorf and Ganopolski 1999; IPCC 2007; McKenney et al. 2007). The likely scenarios of future climate for the boreal regions of North America include higher temperature, longer growing season, and more frequent climate extremes such as prolonged drought (McKenney et al. 2007; IPCC 2012). As long-lived organisms, boreal forest tree species may face considerable challenges in adapting to changes in climate that are expected to be more rapid than those that occurred in the past (Savolainen et al. 2007; Johnston et al. 2009).

A potential consequence of climate change for forest ecosystems is the disruption of evolutionary equilibrium between local forest populations and their surrounding environments. Having evolved under local selection pressures, especially those imposed by the colder climate of the postglacial and preindustrial era for thousands of years, forest populations have become more or less adapted to their local climatic conditions (Savolainen et al. 2007; Aitken et al. 2008). Local adaptation of plant and forest tree species across the landscape has resulted in population differentiation of morphological and physiological traits (Skrøppa 1982; Blum 1988; Rehfeldt 1989; Sork et al. 1993; Li et al. 1997; Wu and Ying 2004) that may be detected by functional gene markers (Namroud et al. 2008; Fournier-Level et al. 2011). Knowledge and observations about the evolutionary process of local adaptation have established the principle that “local seed is best,” which is often explicitly or implicitly assumed in delineating seed zones for tree species to guide artificial forest regeneration (Campbell 1979; Rehfeldt 1989; Parker 1992). Unfortunately, a rapidly changing climate and the adaptation “lags” of forest tree populations resulting from their long lifespan and limited gene flow distance (Aitken 2000; Young et al. 2000) may have disrupted the rate of synchronization. Results from provenance trials of tree species have suggested that: (1) at least for quantitative traits such as growth rate, local climatic conditions are becoming suboptimal for some forest populations; and (2) the potential for enhanced land productivity resulting from a warmer climate in boreal forest regions will not be fully realized by local tree populations (Schmidtling 1994; Carter 1996; Morgenstern et al. 2006; Wang et al. 2006; Rweyyongeza et al. 2011).

Intraspecific assisted migration (ISAM) (Leech et al. 2011), a forest management practice that guides tree seed transfer within a species' natural range during artificial forest regeneration, can be an effective strategy to enhance forest adaptation to climate change, as well as to increase forest productivity (Johnston et al. 2009). Abundant information suggests that forest species with widespread natural distribution have large natural genetic variation in adaptation and growth among geographical natural populations, or *provenances*, which have evolved in synchronization with local climate (Morgenstern and Mullin 1990; Matyas and Yeatman 1992; Li et al. 1997; Morgenstern and Copis 1999). Understanding provenances' adaptation and growth potential in their native growing environments could improve our understanding of the stability and resilience of present forests, while also indicating their vulnerability to climate change. Matching provenances to their optimal growing environments through remapping their geographic deployment zones (Wang et al. 2006; Ying and Yanchuk 2006; Thomson and Parker 2008) could potentially improve the stability and resilience of newly regenerated forests, at least temporarily, depending on the rate of climate change. By enabling the full expression of tree growth potential, and hence higher forest productivity (Johnston et al. 2009), ISAM may also result in greater rates of carbon sequestration and ecological resilience in boreal forests (Leech et al. 2011).

Long-term rangewide provenance trials are a valuable source of information for understanding vulnerability to impacts from climate change and the development of ISAM strategies (Matyas 1994; Carter 1996; Wang et al. 2006). Data from rangewide provenance tests reveal landscape-level intraspecific genetic variability and its spatial trends. Additionally, multiple field trial locations under varying climatic conditions, particularly long-term data from older trials, can enhance data reliability in revealing the both vulnerability and adaptive capacity (such as to frost and drought) of individual provenances and their interactions with climate of existing forests and of planting sites using ISAM. The use of such information for ISAM has been demonstrated using provenance *response* and *transfer functions* (Rehfeldt et al. 1999; Wang et al. 2006, 2010; Hamann et al. 2011). Because of the high value of information from provenance test data in guiding assisted migration, trials involving wide range provenances (from California to the Yukon) of 18 commercially important species were recently established in multiple locations along a broad geographic region and climatic gradient (O'Neill et al. 2013).

White spruce (*Picea glauca* [Moench] Voss) is one of the few boreal conifer species with a transcontinental natural distribution in North America (Nienstaedt and Zasada 1990). In Ontario, Canada, white spruce is a major component of natural forests and provides many products of economic value to the province (Ontario Ministry of Natural Resources [OMNR], 2006). During the late 1970s and early 1980s, the Canadian Forest Service, in cooperation with OMNR, established the 410-series of white spruce rangewide provenance tests, which included 16 field trial locations within Ontario (Morgenstern and Copis 1999). Early results from this series were previously reported for individual trials or provenances (Morgenstern and Copis 1999; Cherry and Parker 2003; Lesser et al. 2004; Morgenstern et al. 2006). In this study, we examine large-scale spatial patterns of genetic variation in adaptation and growth indicators of white spruce provenances across all 16 trials. We anticipate the usefulness of such information in: (1) assisting the development of an ISAM strategy to adapt white spruce to climate change within and outside Ontario and (2) assessing vulnerability of existing forests to climate change, and (3) contributing to policies for conserving genetic resources of this species.

## Materials and Methods

### Provenance samples & field trial establishment

**Figure 1:**
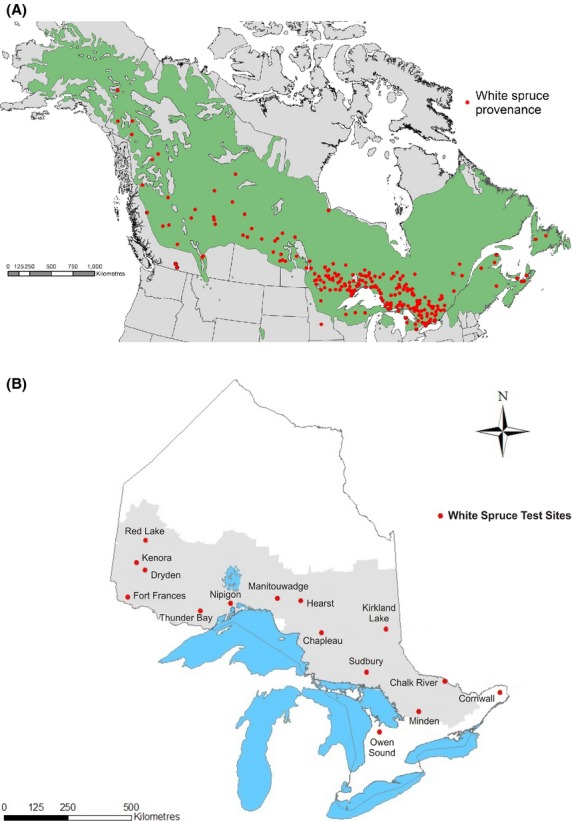
Locations of (A) white spruce provenance origin and (B) field trial sites in Ontario used in the white spruce 410-series rangewide test. Green shading in map (A) indicates the natural range of white spruce distribution and gray shading in map (B) indicates the managed forest area in Ontario.

Provenance seed was sampled from the natural range of white spruce across North America between 1972 and 1976; and 245 provenances were included in the 410-series of experiments across Ontario sites. The objectives of the 410-series were to assess genetic variation across the entire range of white spruce and to examine within-region genetic variation for selected areas, including Ontario (Morgenstern and Copis 1999) (Fig. 1A). Detailed information about the individual provenances was published by Morgenstern and Copis (1999).

Between 1978 and 1985, 16 field trials in the 410-series were established within Ontario. Although additional field trials of this series were established in other eastern Canadian provinces, data from these locations were not available for inclusion in our study. Field trial sites in Ontario varied in soil depth and texture and occurred along a climatic gradient (Fig. 1B) that encompassed the provincial range of white spruce growing environments. Summary information, including climate norms (1971–2000) (obtained using the SEEDWHERE software of McKenney et al. 1999) and experimental design for the Ontario trial locations, is presented in Table 1. All trial areas were mechanically site prepared prior to planting. The age of planting stock varied from 1 to 3 years among trials and postplanting (1–2 year) tree survival rate was uniformly high (≥94%; Table 1). The experimental design for each field trial was a randomized complete block design with multiple-tree plots. These were replicated 5–8 times and the number of trees per provenance plot within an experimental block varied from 4 to 10 among the trials (Table 1). No field trials contained all 245 provenances. Rather, each field trial tested a subset of 48–86 provenances. While many of the common provenances overlapped between pairwise trials (Table 2), 105 of the 245 provenances were tested in fewer than four Ontario field trials and only two provenances were tested across all 16 trials.

**Table 1:** Location details, composition, and initial survival for the 410-series white spruce provenance trials in Ontario

Trial location	Lat. (°N)	Long. (°W)	Elev. (m)	MAT (°C)	MAP (mm)	GSL (day)	Tree age	No. of provenances	No. of replications	Trees/plot	Post-planting survival (%)
Cornwall	45°22′	74°44′	24	5.57	1011	205	23	64	6	8	98.0
Chalk River	45°59′	77°26′	170	4.62	846	193	20	71	8	4	–
Chalk River	45°59′	77°26′	170	4.62	846	193	20	71	8	4	–
Minden	45°00′	78°53′	376	4.78	1090	193	25	80	5	10	99.0
Kirkland Lake	48°00′	80°20′	308	1.73	839	172	19	80	5	10	97.0
Owen Sound	44°24′	80°55′	200	6.29	1058	211	22	64	6	8	98.0
Sudbury	46°31′	81°24′	350	3.72	878	185	24	86	5	9	97.0
Chapleau	47°59′	83°42′	451	1.13	888	165	25	80	5	10	97.0
Hearst	49°04′	84°53′	320	0.39	789	161	21	85	5	9	94.0
Manitouwadge	49°12′	86°01′	300	0.92	803	165	26	80	6	10	96.0
Nipigon	48°58′	88°32′	190	1.38	724	165	26	80	6	8	98.0
Thunder Bay	48°38′	90°09′	475	1.56	753	161	26	78	6	5	98.0
Dryden	49°54′	93°20′	410	1.88	681	176	26	78	5	10	99.0
Red Lake	50°56′	93°29′	370	1.19	651	173	21	80	5	9	98.0
Kenora	50°08′	93°50′	410	1.86	657	177	28	48	5	5	99.0
Fort Frances	48°45′	93°58′	340	2.85	686	181	28	65	5	5	99.0

Lat., long., MAT, MAP, and GSL are latitude, longitude, mean annual temperature, mean annual precipitation, and growing season length, respectively. Two trials were conducted in Chalk River.

**Table 2:** Numbers of common provenances of white spruce represented between paired field trial locations

			No. of common provenances														
			
Trial location	Total no. of provenances	Trial no.	2	3	4	5	6	7	8	9	10	11	12	13	14	15	16
Cornwall	64	1	30	29	46	43	28	34	42	25	32	25	37	25	25	14	17
Chalk River G1	71	2		70	31	27	22	46	30	53	26	31	24	29	21	9	13
Chalk River G2	71	3			31	28	21	45	29	54	25	31	23	28	21	9	13
Minden	80	4				30	39	43	61	26	38	27	43	27	28	17	21
Owen Sound	67	5					24	28	28	37	25	28	27	26	25	15	18
Kirkland Lake	80	6						31	50	30	44	44	36	34	24	14	18
Sudbury	86	7							47	53	29	29	28	26	25	14	19
Chapleau	80	8								29	40	31	41	28	27	15	20
Hearst	85	9									29	38	25	34	28	10	16
Manitouwadge	80	10										60	68	52	39	25	29
Nipigon	80	11											52	55	32	17	22
Thunder Bay	78	12												52	39	26	32
Dryden	78	13													42	24	32
Red Lake	80	14														40	55
Kenora	48	15															48
Fort Frances	65	16															

Two trials were conducted in Chalk River.

Trials were assessed in the summer of 2001 at tree ages from 19 to 28 years and measurements included individual tree height, diameter at breast height (DBH), and tree health status (mortality and mechanical damage). Individual tree stem volume was subsequently estimated using a taper equation (Popovich 1972) developed using data from white spruce plantations grown in Québec, Canada. This equation was independently validated and found to produce unbiased results with high accuracy for white spruce grown in plantations in the upper Great Lakes region of the United States (Rauscher and Harding 1993).

### Predicting provenance effects

The experimental data were largely balanced within each of the individual trials. However, because individual trials included only a subset of provenances, the data became highly imbalanced when the 16 trials were pooled to perform a combined analysis, a procedure necessary to evaluate the relative performance of all 245 provenances. In addition to the differing subsets of provenances, data imbalances resulted from differences among field trials in: (1) age of planting stock (1–3 years), (2) tree age at measurement (19–28 years), (3) number of replications per trial (5–8), and (4) number of trees per plot (4–10 trees). Additionally, varying climate, soil conditions, competing vegetation, and damage by insects and frost across trial locations (Morgenstern and Copis 1999) resulted in considerable differences in mean tree sizes among field trials. As such, the marginal means of provenance performance across trials were biased and poor indicators of the intrinsic growth potential of the provenances.

Data standardization is effective in removing scale effects caused by tree size differences in tree genetic evaluation (White and Hodge 1989), thereby satisfying the underlying homogeneous variance assumptions often associated with analytical linear models (Lynch and Walsh 1998; White et al. 2007). In tree genetic data analysis, data standardization is recommended because it does not alter analytical outcomes in either estimating genetic parameters (such as heritability and genetic correlations) or genotype ranking (White et al. 2007). In this study, prior to statistical analysis, data were standardized to transform observations of individual trials with the same mean and variance (which were taken from the summary statistics of pooled data). Because larger phenotypic variance is often associated with larger tree size (White and Hodge 1989), the means and variances were consequently compressed after data standardization for trials of above average size and expanded for trials of below average size.

To evaluate the performance of all 245 rangewide provenances, which were directly or indirectly connected through overlapping common provenances across the 16 field trials (Table 2), a mixed linear model (eq. 1) was used in which the effects of provenances and their interactions with trial locations were treated as random, while trial location and blocks within trials were treated as fixed. In matrix notation, the analytical model was:


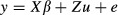
(1)

where *y* is the vector of observations of individual trees; *X* is the incidence matrix of fixed effects included in vector *β* (e.g., trial locations and blocks within a trial); *Z* is incidence matrix of random effects in vector *u,* which comprises provenance and the interaction between provenance and trial location; and *e* is the vector of residuals. Henderson's mixed linear model equation (Henderson 1984) has provided the theoretical basis for simultaneously yielding best linear unbiased estimation (BLUE) of fixed effects and best linear unbiased prediction (BLUP) of random provenance effects (Searle et al. 1992; Lynch and Walsh 1998; White et al. 2007) for mixed models like equation 1. In this study, this analytical approach permits the utilization of the genetic variance/covariance matrix of provenances across field trials to improve evaluation of provenance effects. The SAS *Proc Mixed* procedure (SAS Institute 2011) was used to implement the BLUP analysis with standardized data for continuous traits (e.g., tree height, DBH & volume) and the *Proc Glimmix* procedure was used for binary trait (e.g., survival), respectively.

### Modeling geographic patterns and provenance response

The BLUPs of provenance effects were modeled with the geographic coordinates of provenance origins (i.e., latitudinal & longitudinal coordinates) to reveal large-scale spatial patterns of variation in growth and adaptation, which may have differentiated under the influence of local climate in the past. Polynomial curves with the appropriate orders were used to approximate the distribution patterns of data points.

Because most provenances were tested in less than one-third of the field trials, and each field trial included only a subset of the 245 provenances (Table 2), it is less informative to examine the response of individual provenances to climatic conditions of the trial sites. Instead, the growth response by regional provenance groups was examined, with each group representing a geographic area from which a relatively large number of provenances (*n* ≥ 63) were sampled. Specifically, provenances were assigned to southern (lat. <47°N, *n* = 84), central (47°N ≥ lat. <50°N, *n* = 94), and northern (lat. ≥ 50°N, *n* = 63) regional groups (four provenances were not assigned because of missing geographic coordinates). Although the boundaries of these regional provenance groups were determined arbitrarily, efforts were made to ensure that provenances within a regional group exhibited similar survival and growth patterns. Our exploratory analysis indicated that this classification of regional provenance groups resulted in a clearer delineation of the response patterns to climatic conditions of the trial sites than a classification from a multivariate regression tree analysis (Hamann et al. 2011).

Analysis of regional groups at each of the 16 trial locations provided a simple but insightful indication of the response of white spruce to climatic conditions. For this analysis, the relative growth of provenance groups (expressed as ratio of provenance group mean to the overall trial mean) was plotted against some commonly used thermal indicators of planting site climate, that is, mean annual temperature (MAT), growing season length (GSL), and latitude.

### Developing a universal response function

Wang et al. (2010) proposed a multiple regression approach to use climatic variables to predict population performance across test sites, and named the predictive equation a universal response function (URF). Conceptually, climate variables from the site of provenance origin can be superior to geocoordinates in predicting provenance response to climatic conditions at planting sites, because climate at provenance origin could contribute an evolutionary force to cause genetic differentiation and local adaptation (Aitken et al. 2008). Analytically, the URF approach uses climatic variables at provenance origin and those at planting site as independent prediction variables (as well as their quadratic effects and interactions), with population performance (such as mean tree height growth) as the dependent variable (Wang et al. 2010). Using stepwise selection in a multiple regression analysis, the URF may be able to identify influential climatic variables explaining provenance growth variation across planting sites. In this study, we followed Wang et al. (2010) in developing a URF for white spruce based on the 410-series provenance tests data. Climatic variables of both provenance origins and planting sites were generated using SEEDWHERE software (McKenney et al. 1999). Specific climatic variables at provenance origin we included in the analysis included mean annual temperature (MAT_p)(°C), January minimum temperature (JMT_p)(°C), February minimum temperature (FMT_p)(°C), growing season length (GSL_p)(days), annual precipitation (AP_p)(mm), and precipitation of the driest period (PDP_p)(mm). Climatic variables at planting sites were mean annual temperature (MAT_s)(°C), mean annual minimum temperature (MAMT_s)(°C), growing season length (GSL_s)(days) and annual precipitation (AP_s)(mm). The quadratic effects and interactions between the above variables were generated as the product of a climatic variable with itself or with another variable. In total, 43 independent variables were used in model building.

Provenance mean height growth at individual field trials was used as the dependent variable. Because field trials differed in age at measurement (Table 1), an adjustment was made to approximate provenance mean height growth at a standard age of 25 years across field trials. This was estimated by using Goelz and Burk's (1992) base-age invariant site index model, which was parameterized for white spruce by Payandeh and Wang (1995) as:


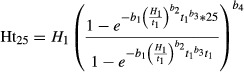
(2)

where Ht_25_ is the estimated provenance height at age 25, *H*_1_ is provenance height measured at the age *t*_1,_ *e* is the base of natural logarithm and *b*_1_–*b*_4_ are model parameter estimates with the values of 5.7336, 0.8236, −1.880 and 2.7703, respectively. The SAS *Proc Reg* procedure with the stepwise option (SAS Institute 2011) was applied for variable selection. The *P* values for independent variables to enter and stay in the model were set at 0.15 and 0.05, respectively.

## Results

### Provenance survival

**Figure 2:**
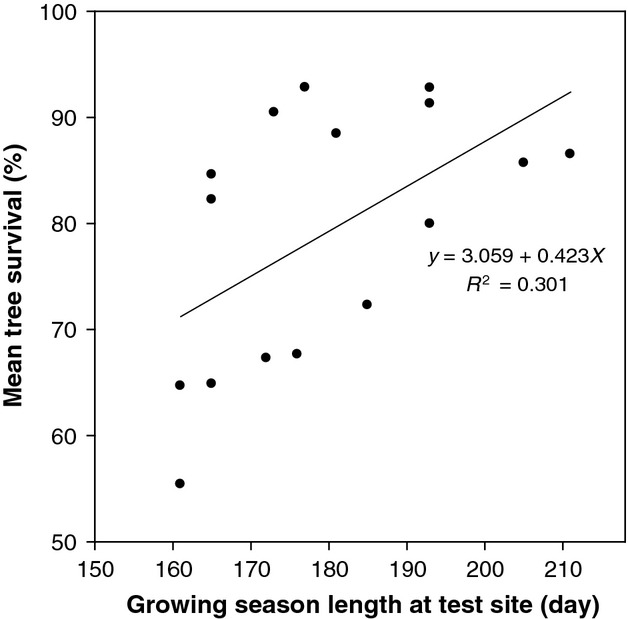
The relationship of provenance survival with growing season length. The linear regression equation and coefficient of determination (*R*^2^) are presented.

Initial survival was uniformly high (≥94%) across the 16 field trials, but then declined. At the time of measurement in 2001, survival was <70% at five trial sites (i.e., Kirkland Lake, Hearst, Manitouwadge, Thunder Bay, and Dryden), remained above 80% for 10 sites, with the remaining site (Sudbury) at 72% (Table 3). While individual trees were not assessed to define specific causes of mortality (e.g., pests, frost/cold damage, competition), survival rates were weakly correlated with mean annual temperature (MAT) (*P* < 0.07) and significantly affected by growing season length (GSL) (*P* < 0.03; Fig. 2), with shorter GSL and lower MAT associated with lower survival rates.

**Table 3:** Survival and mean tree height, diameter at breast height (DBH) and stem volume at time of measurement for 16 white spruce provenance trials in Ontario

		Height (m)		DBH (cm)		Volume (m^3^)	
				
Trial location	Survival (%)	Mean	SD	Mean	SD	Mean	SD
Cornwall	85.7	6.90	1.85	11.51	3.87	0.064	0.067
Chalk River G1	92.7	5.95	1.32	10.26	2.98	0.035	0.032
Chalk River G2	91.2	5.57	1.31	9.45	2.79	0.026	0.025
Minden	80.0	6.08	1.88	7.88	2.94	0.021	0.027
Kirkland Lake	67.3	2.89	1.18	3.51	2.15	0.001	0.002
Owen Sound	87.0	3.46	1.00	4.67	1.65	0.003	0.006
Sudbury	72.3	5.04	1.62	6.70	2.79	0.012	0.016
Chapleau	82.2	6.88	1.63	8.92	2.66	0.028	0.027
Hearst	64.7	5.66	1.41	9.36	3.28	0.028	0.028
Manitouwadge	64.6	3.39	1.45	5.12	3.19	0.007	0.014
Nipigon	84.6	4.42	1.19	6.72	2.06	0.008	0.009
Thunder Bay	55.8	7.20	1.54	9.93	3.00	0.040	0.038
Dryden	67.6	2.88	1.01	3.76	1.68	0.001	0.003
Red Lake	90.4	4.32	1.14	7.51	2.61	0.011	0.012
Kenora	92.7	6.53	1.43	9.10	2.40	0.026	0.022
Fort Frances	88.4	4.28	1.40	6.08	2.59	0.008	0.011

SD is the standard deviation of measurements within a trial. Two trials were conducted in Chalk River.

**Figure 3:**
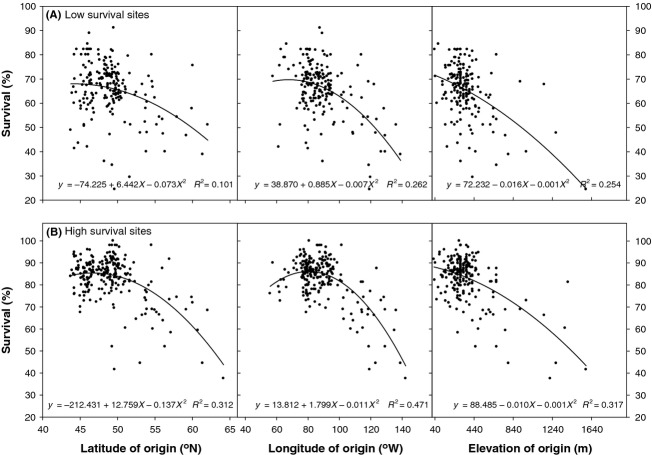
Relationship of provenance survival with latitude, longitude, and elevation of provenance origin for trial locations with survival of (A) (<70%; *n* = 5) and (B) (>72%; *n* = 11). Quadratic polynomial models and coefficients of determination (*R*^2^) are presented.

The relationship of survival rate with latitude, longitude, and elevation of provenance origin was examined to elucidate survival differences among provenances (Fig. 3). Across the five test sites with low survival, which involved 196 provenances, the latitudinal, longitudinal, and elevational trends were delineated by statistically significant quadratic polynomial curves (Fig. 3A). Provenances from southern Ontario and southwestern Québec (i.e., lat. <47°N) survived as well as local provenances originating close to the trial locations (i.e., lat. 47°–50°N; long. 85°–94°W) (*P* > 0.63), and significantly better than provenances from western Canada and Alaska (i.e., lat. > 50°N) (*P* < 0.004). For the 11 trials with higher survival rates (72–93%), the 241 provenances planted showed patterns of survival (Fig. 3B) similar to the lower survival trials (Fig. 3A), although latitudinal and longitudinal polynomial curves explained a greater portion of the variation in survival among provenances (Fig. 3A and B). These quadratic polynomial curves approximated the survival data better than linear lines, with 5–44% increase in *R*^2^ of model fitting although the improvement was not always statistically significant. Combined, these results indicated that across the field trials, provenances from southern Ontario and southwestern Québec demonstrated similar adaptability to those from northern Ontario and eastern Canada and significantly higher adaptability than those from western Canada.

When field trials were grouped into relatively harsh (i.e., MAT < 3°C; 10 trials) and relatively mild (i.e., MAT ≥ 3°C; 6 trials) thermal environments, survival rates of provenances from south-central Ontario and southwestern Québec (i.e., 44°–47°N; 74°–85°W) were again similar to those from northern Ontario and eastern Canada (i.e., 47°–50°N) in the harsh environment; both had significantly better survival (*P* < 0.004) than provenances from western Canada and Alaska. In the milder environment, white spruce provenances from south-central Ontario survived similar to those from northern Ontario and eastern Canada (87 vs. 85%; *P* < 0.10); and again survived significantly better than provenances from western Canada and Alaska (85–87 vs. 82%; *P* < 0.0001).

### Provenance growth potential

**Figure 4:**
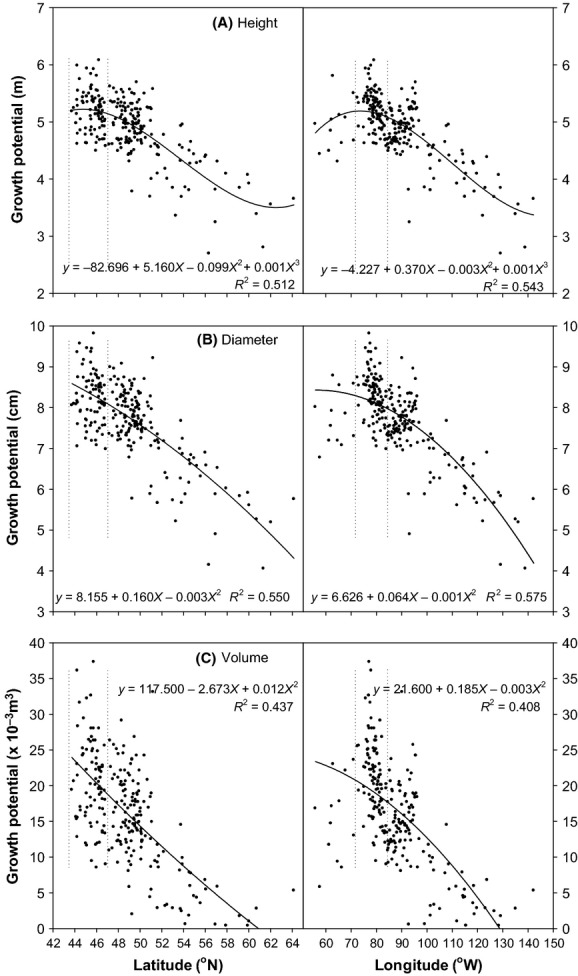
Relationship of (A) tree height, (B) stem diameter, and (C) stem volume growth of provenances with latitude and longitude of provenance origin. The area between the two vertical lines represents the geographic region of provenances from south-central Ontario and southwestern Québec associated with superior tree growth. Polynomial models and coefficients of determination (*R*^2^) are presented.

Tree growth varied considerably across the 16 trial sites, reflecting differences in climate, soil, tree age, etc. (Table 3). After removing inter-trial tree size variation using standardized data, the BLUPs of provenance effects on tree height, DBH, and stem volume varied from 2.70 to 6.08 m (mean 4.9 m), 4.1 to 9.8 cm (mean 7.7 cm), and 0.001 to 0.037 m^3^ (mean 0.017 m^3^), respectively, among the 245 rangewide provenances tested. Similar to survival, height, DBH, and stem volume for the rangewide provenances were related to their geographic origins, which were slightly better approximated by quadratic or cubic polynomial curves (Fig. 4) than linear lines, with up to 15% increase in R-square in model fitting (although the improvement may not be statistically significant). Provenances from south-central Ontario and southwestern Québec exhibited the highest growth rates, followed by provenances from northern Ontario and eastern Canada. Provenances from western Canada and Alaska exhibited substantially lower growth rates than those from other regions (Fig. 4; Table 4).

**Table 4:** Mean tree height, diameter, and stem volume of three regional provenance groups of white spruce trees measured in 2001

Regional group (No. of provenances)	Height (m)	DBH (cm)	Volume (m^3^)
Southern (84)	5.19^a^	8.25^a^	0.020^a^
Central (94)	5.02^b^	7.95^b^	0.017^b^
Northern (63)	4.41^c^	6.78^c^	0.010^c^

DBH, diameter at breast height.

The three regional provenance groups are southern (lat. < 47°N), central (47°N ≥ lat. <50°N), and northern (lat. ≥ 50°N). Mean values with different superscript letters differ significantly (*P* < 0.001) according to Tukey's multiple comparison test.

### Response of provenance groups to trial site climate

**Figure 5:**
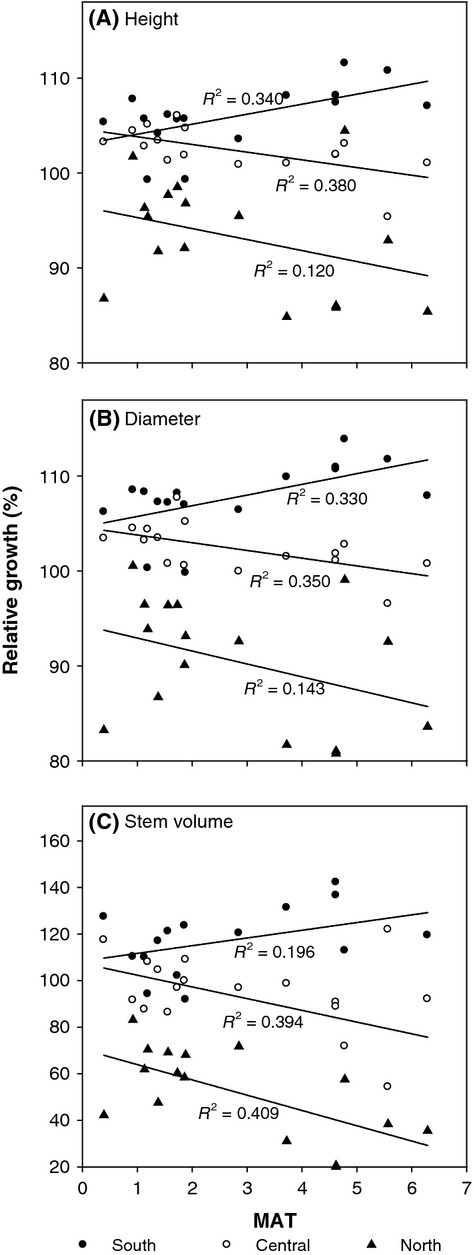
Relative (A) height, (B) diameter, and (C) stem volume growth of three regional provenance groups to trial means as related to the mean annual temperature (MAT) of the field trial locations. The three regional provenance groups are southern (lat. < 47°N, *n* = 84), central (47°N ≥ lat. <50°N, *n* = 94) and northern (lat. ≥ 50°N, *n* = 63), where n is the number of provenances included. Coefficients of determination (*R*^2^) for linear regression equations are presented.

The three regional provenance groups exhibited contrasting, distinct trends in response to MAT of field trial locations (Fig. 5). In general, the southern group of provenances exhibited the highest relative growth at the warmest sites. This relative growth superiority decreased gradually as MAT decreased across trial locations. When planted further north, where MAT was close to 1°C, the growth of southern provenances was similar to that of local provenances. Growth performance of provenances in the central group, mostly from northern Ontario and eastern Canada, showed the opposite trend. Their growth was comparable with the southern group at northern sites but became increasingly inferior at warmer trial locations. Provenances in the northern group, generally associated with higher latitude and altitude of origin, showed a parallel growth response to that of the central group, but with the intercept of the response line shifted substantially downward (Fig. 5), indicating much lower growth potential. Provenance group-by-trial location interaction was evident, which mainly reflected the relative differences among provenance group means because their rankings remained unchanged.

Other thermal indicators of field trial sites, such as GSL and latitude, were highly correlated with MAT, and the relationship trends of growth responses of provenance groups with these indicators were similar (data not shown).

### The universal response function (URF)

Among the 43 candidate climatic variables used to predict provenance mean height growth across field trials, eight were selected into the URF (cumulative *R*^2^ = 0.60) (Table 5), with six of them representing the climatic conditions of field trial locations and two representing interactions between climatic variables at trial locations and at provenance origins. While Mallow's Cp statistic suggested that provenance growth difference could be further explained by adding independent variables, there was no other climatic variables that could stay in the model at the significance level of *P* < 0.05. The six climatic variables at trial locations were mean annual temperature (MAT_s), mean annual minimum temperature (MAMT_s), annual precipitation (AP_s) and their quadratic effects (i.e., MAT_s^2^, MAMT_s^2^ and AP_s^2^). Predictive climatic variables at provenance origins were mean annual temperature (MAT_p) and February minimum temperature (FMT_p), which interacted with climatic variables at trial locations (i.e., MAT_p × AP_s and FMT_p × GSL_s). In the established URF, no single climatic variable was predominantly predictive of provenance performance as shown by the partial *R*^2^s; however, selected climatic variables for trial locations have jointly explained the majority of the growth variation across field trials, with a cumulative partial *R*^2^ of 0.539 (Table 5). The interaction between annual mean temperature at provenance origin and annual precipitation at field trial site (MAT_p × AP_s) indicated that a combination of higher MAT_p and greater AP_s could have a positive effect on provenance growth, while the interpretation for the interaction between February mean minimum temperature at provenance origin and the growing season length at field trial site (FMT_p × GSL_s) was not straightforward. Nevertheless, these two interaction terms in the URF accounted for only 0.063 cumulative partial *R*^2^.

**Table 5:** Multiple regression analysis of provenance mean tree height growth across field test against climatic variables at test locations and provenance origins

Independent variable	Variable domain	Parameter estimate	Partial *R*^2^	Model *R*^2^	*C*(p)	*F*	*P*
Intercept		−44.27					
MAT_s^2^ (°C)	0.15–39.56	2.41233	0.0740	0.0740	1616.91	93.95	<0.0001
MAMT_s (°C)	−5.96 to 1.51	−10.22872	0.0690	0.1431	1410.91	94.58	<0.0001
MAMT_s^2^	0.12–35.52	−1.20823	0.1492	0.2922	963.475	247.22	<0.0001
MAT_s (°C)	0.39–6.29	−9.92411	0.1127	0.4049	626.001	221.90	<0.0001
AP_s^2^	423801–1188100	−0.0000545	0.0606	0.4655	445.404	132.78	<0.0001
AP_s (mm)	651–1090	0.08333	0.0737	0.5392	225.465	187.02	<0.0001
MAT_p × AP_s	−3222 to 7706	0.00074057	0.0257	0.5649	149.943	69.13	<0.0001
FMT_p × GSL_s	−5389 to −1360	−0.00146	0.0376	0.6025	38.6002	110.54	<0.0001

MAT, MAMT, AP, FMT, GSL, are mean annual temperature, mean annual minimum temperature, annual precipitation, February minimum temperature and growing season length, respectively, with _p or _s indicating climatic variable at provenance origins or trial site location. *C*(*p*) is Mallow's *Cp* statistic.

## Discussion

The 410-series rangewide provenance test represented a large-scale historical effort in Canada to understand the intraspecific genetic variability of white spruce growth and adaptation (Morgenstern and Copis 1999). This study is the first to compare adaptation and performance for rangewide provenances across 16 field trials established in Ontario.

### Provenance adaptation

The adaptability of white spruce provenances to different growing environments was mainly indicated by survival. Unfortunately, the causes of tree mortality were not assessed in this long-term provenance test. Field trials with lowest survival were located in northern Ontario where winters are coldest. The statistically significant association between mean survival rate and GSL at a trial location suggests that thermal regime and low temperature are factors influencing survival in northern Ontario.

Southern provenance survived equally well as local provenances did at northern planting sites, while provenances whose origins were north of the northern planting sites had significantly poorer survival. This result is contrary to the general view that tree species populations from milder environments will suffer at sites north of their origin because they are less cold hardy than those originating from more northern, colder environments (Matyas and Yeatman 1992). However, white spruce, as a boreal tree species, can tolerate very low winter temperatures (<−40°C) through the mechanisms of “deep undercooling” or “extra organ freezing” (Sakai 1979, 1983; Bannister and Neuner 2001) when trees are dormant. Thus, minimum winter temperature in northern Ontario is unlikely to cause mortality of white spruce. Southern Ontario white spruce provenances also survived and grew well in central Alberta where winter climatic conditions were cold as or colder than those of the northern Ontario 410-series trial locations (Rweyyongeza et al. 2011). Therefore, as shown by our results, almost all the white spruce provenances can survive the historical winter climate in northern Ontario while in winter dormancy.

In contrast to minimum winter temperature, relatively mild freezing in spring and fall may damage or kill young white spruce. Fall frost damage is relatively infrequent and less consequential to white spruce (Nienstaedt and Zasada 1990). Spring frost is more frequent and more damaging to white spruce than to other spruces, such as black (*Picea mariana* [Mill.] BSP) and red spruce (*Picea rubens* Sarg.), as white spruce requires lower thermal sums to initiate bud flush (e.g., O'Reilly and Parker 1982; Blum 1988). Spring frost damage to white spruce can be particularly high when warm spring temperatures induce earlier than normal dehardening and bud burst (Clements et al. 1972; Man et al. 2009). However, conifer species of more southerly origins often require higher thermal sums to flush buds than those from northerly populations (Ekberg et al. 1994; Partanen and Beuker 1999; Søgaard et al. 2008). Southern white spruce provenances were found to delay bud flushing for several days (Blum 1988) and were therefore cold hardier than more local northern provenances under the same thermal regime in the spring (Simpson 1994).

Thus, at northern Ontario trial locations, the southern provenances tested in our study may have suffered less mortality from low temperatures than the northern provenances due to avoidance of spring frost by slightly delayed bud break (Blum 1988; Simpson 1994).

### Provenance growth

The relationship of predicted height, DBH, and volume growth with latitude of provenance origin indicated a decreasing trend of growth potential as latitude at origin increased (Fig. 4). Along a longitudinal gradient, provenance performance increased slightly as provenance origins moved from eastern Canada to Ontario (i.e., from 55° to 72°W), peaked in Québec and southern Ontario (between 72° and 85°W), and then decreased markedly further west. These latitudinal and longitudinal trends are consistent because latitude and longitude are highly autocorrelated in the range of white spruce distribution (Fig. 1A) (Nienstaedt and Zasada 1990). That is, latitude of provenance origins decreased from eastern Canada to southwestern Québec and southern Ontario and then increased toward to the west coast (Fig. 1). Both the latitudinal and longitudinal patterns identified southwestern Québec and southern Ontario as the source of provenances with the best overall performance. These patterns of growth variation of white spruce provenances are consistent with results from other studies in Canada and the United States (Khalil 1985; Nienstaedt and Zasada 1990; Cherry and Parker 2003).

Considerable differences in growth were evident among white spruce provenances tested at sites across Ontario. The best performing provenances exhibited nearly double the growth in height and DBH of the worst growing provenances. The superior growth of provenances from south-central Ontario and southwestern Québec is consistent with results for several trial locations scattered across the range of white spruce (Nienstaedt 1969; Khalil 1985; Li et al. 1993, 1997; Rweyyongeza et al. 2011). These populations typically outperform local provenances at test sites located at large distances from their geographic origin. For example, Rweyyongeza et al. (2011) reported that at the age of 24 years, white spruce provenances from southern Ontario and Québec had the highest growth rate in central Alberta, compared with local provenances and those from western and eastern Canada. Our results suggest that seed sources of white spruce native to northern Ontario and Quebec will grow relatively more slowly, compared with more southerly provenances, as temperatures warm. Provenances native to western Canada and Alaska may be even more severely affected by climate warming within their home range, with relative volume growth decreasing by as much as 50% for a mean annual temperature increase by 5°C, compared with southerly provenances. We cannot project how white spruce in south-central Ontario and southwestern Québec will respond to climate warming, as the trial lacks test sites at locations that are warmer than those in this part of the species' range. These results confirm recommendations that their use for planting may increase forest productivity and carbon sequestration, and indicate that these populations represent a promising candidate genetic base for ISAM of white spruce through its northern range.

### Predicting provenance performance with URF

Based on the derived URF, white spruce provenance mean height growth across field trials was partially predicted by a combination of climatic variables describing field trial locations and provenance origins. The predictability (based on *R*^2^) of this study's URF was 0.60, compared to 0.81 found by Wang et al. (2010). Several factors could have contributed to the poorer quality of the URF in the present study, including: (1) potentially high variability in soil fertility among trial sites that affected mean tree height growth independent of climate. This is suggested by field trials which are spatially close and have similar climatic conditions differing considerably in overall mean tree height growth (for example, Owen Sound vs. Minden, and Dryden vs. Kenora), (Tables 1 and 3); and (2) age differences among trials at time of measurement (Table 3). Although adjustment was made to account for age differences, we cannot rule out the possibility that height adjustments of provenances not present at all sites was imprecise or insufficient. In addition, the highly predictability of the URF in the lodgepole pine (*Pinus contorta*) study (Wang et al. 2010) could have benefited from uniform trial age and soil conditions among that study's field trials.

We interpret the finding that the independent prediction variables selected into the URF represented mostly climate at field trial locations to mean that URF primarily indicates phenotypic plasticity of white spruce to climate. The absence of a strong relationship between provenance performance and climate at provenance origin indicates that climatic selection pressures on white spruce were not important in causing provenance genetic differentiation. The URF from this study thus seemed to have limited utility in guiding ISAM.

### Implications for climate change impacts and an intraspecific assisted migration strategy

The results of this study suggest that climate change will cause white spruce populations in northern parts of the species' range to grow relatively slowly, compared with southerly provenances. If the goal of implementing an ISAM strategy is to reduce climate maladaptation, this study indicates there is relatively low risk of mortality of southern white spruce populations planted in colder northern Ontario locations. The higher survival rates of southern provenances at northern sites after 19–28 years suggests that thermal conditions in northern regions (i.e., 47° ≤ lat. < 50°N) were within their cold tolerance capacity. While recognizing the potential for increased disturbance by fire, insects, and disease in boreal forests as a result of a warming climate, a benefit may be higher growth and productivity of some boreal species and populations (Huang et al. 2010). If climate change does not dramatically increase moisture stress as predicted for some boreal regions of North America (IPCC 2012), growth rates may increase for some tree species due to longer growing seasons and higher photosynthetic rates under warmer temperatures (Pastor and Post 1988; Goldblum and Rigg 2005). Growth in northern regions may be further increased by regenerating southern provenances of white spruce that showed improved growth in northern Ontario (Fig. 5). For example, at an average site in northern Ontario with MAT of 1.5°C, over the past 30–40 years provenances from the southern group (moved northward by about 3° latitude) would have produced about 13.5% more mean stem volume than the local populations (i.e., central group) (Fig. 5). An estimated additional 8% increase in stem volume would have occurred under a 1°C increase in MAT. Indeed, it appeared as though the growth of northern white spruce populations is becoming, if not the case already, suboptimal to a warming climate.

Conserving genetic resources of white spruce in south-central Ontario and southwestern Québec is of high ecological and economic importance for adapting this species to climate change. The gene pool of white spruce populations in this region seems to produce the most productive genetic material to enhance climate change adaptation through ISAM in Ontario and other regions where white spruce is grown. Very importantly, it appears that such relocations could take place now, since climate conditions during the 20–30 years as these trials were established did not result in freezing damage to southern populations planted at northern sites.

Genetic and physiological mechanisms linked to superior growth and adaptability in diverse climatic habitats are largely unknown and information (such as genetic diversity and allele richness) is lacking from direct comparisons between the southern and northern populations of white spruce. Pollen and macrofossil data and recent analyses of chloroplast DNA suggest that postglacial expansion of white spruce from two refugia may have resulted in genetically distinct white spruce populations across eastern Canada and the United States (Ritchie and MacDonald 1986; Anderson et al. 2006; de Lafontaine et al. 2010). White spruce is believed to have spread from an Appalachian (Pennsylvania) refugium into New England, the Altantic Provinces, and Québec, while the Mississippian (Kansas, Iowa, Missouri, Illinois) refugium migrated to colonize the Great Lakes basin, northern Ontario, and westerly into central Canada (Ritchie and MacDonald 1986; de Lafontaine et al. 2010). We propose that the founder populations of white spruce in southern Ontario and southwestern Québec may have originated from migration from both glacial refugia, resulting in a broader genetic base to harbor beneficial alleles associated with superior tree growth and climatic adaptability. The genetic base of white spruce outside this region could have been narrowed by either the selection forces of local adaptation (Aitken et al. 2008) or the founder populations may have arisen from only one of these glacial refugia (Ritchie and MacDonald 1986). If confirmed, this may help explain why white spruce populations in southern Ontario and southwestern Québec are more productive than populations from other regions of the species' natural range. Emerging genomic tools such as high-density functional gene array (Namroud et al. 2008) may eventually provide insight into this phenomenon.
